# Comprehensive investigation of the electronic excitation of W(CO)_6_ by photoabsorption and theoretical analysis in the energy region from 3.9 to 10.8 eV

**DOI:** 10.3762/bjnano.8.220

**Published:** 2017-10-23

**Authors:** Mónica Mendes, Khrystyna Regeta, Filipe Ferreira da Silva, Nykola C Jones, Søren Vrønning Hoffmann, Gustavo García, Chantal Daniel, Paulo Limão-Vieira

**Affiliations:** 1 Laboratório de Colisões Atómicas e Moleculares, CEFITEC, Departamento de Física, Universidade NOVA de Lisboa, 2829-516, Caparica, Portugal; 2 ISA, Department of Physics and Astronomy, Aarhus University, Ny Munkegade 120, DK-8000, Aarhus C, Denmark; 3 Instituto de Física Fundamental, Consejo Superior de Investigaciones Científicas (CSIC), Serrano 113-bis, 28006 Madrid, Spain; 4 Laboratoire de Chimie Quantique, Institut de Chimie Strasbourg, UMR7177 CNRS/Université de Strasbourg 1 Rue Blaise Pascal BP296/R8, F-67008 Strasbourg, France

**Keywords:** cross sections, density functional theory (DFT) calculations, focused electron beam induced deposition (FEBID), photoabsorption, tungsten hexacarbonyl

## Abstract

High-resolution vacuum ultraviolet photoabsorption measurements in the wavelength range of 115–320 nm (10.8–3.9 eV) have been performed together with comprehensive relativistic time-dependent density functional calculations (TDDFT) on the low-lying excited sates of tungsten hexacarbonyl, W(CO)_6_. The higher resolution obtained reveals previously unresolved spectral features of W(CO)_6_. The spectrum shows two higher-energy bands (in the energy ranges of 7.22–8.12 eV and 8.15–9.05 eV), one of them with clear vibrational structure, and a few lower-energy shoulders in addition to a couple of lower-energy metal-to-ligand charge-transfer (MLCT) bands reported in the literature before. Absolute photoabsorption cross sections are reported and, where possible, compared to previously published results. On the basis of this combined experimental/theoretical study the absorption spectrum of the complex has been totally re-assigned between 3.9 and 10.8 eV under the light of spin–orbit coupling (SOC) effects. The present comprehensive knowledge of the nature of the electronically excited states may be of relevance to estimate neutral dissociation cross sections of W(CO)_6_, a precursor molecule in focused electron beam induced deposition (FEBID) processes, from electron scattering measurements.

## Introduction

The electronic structure of tungsten hexacarbonyl, W(CO)_6_, has previously been studied by using a variety of different experimental and theoretical methods, with experiments including vacuum ultraviolet experiments in the wavelength range of 125–350 nm [1–5], and electron energy loss [6–8], photoelectron [9–10], photoionisation [11] and electron momentum [12–13] spectroscopy. In theoretical studies, Dirac-scattered-wave (DSW) calculations [14], molecular orbital energy level calculations [2], relativistic time-dependent density functional theory (TDDFT) calculations [15], and electron momentum spectroscopy calculations [16] have been carried out. Other relevant studies include DFT calculations on the structure of W(CO)_6_ and its behaviour in infrared spectroscopy [17], as well as Raman [18] and infrared [18–20] spectroscopy experiments. Detailed knowledge of the electronic-state spectroscopy of transition-metal hexacarbonyls has attracted particular attention due to the ability of CO to form complexes with metals in low oxidation states. This is possible due to the presence of low-lying empty π*-orbitals, which play a significant role in the stability of carbonyl complexes, and in particular for W(CO)_6_ where the tungsten oxidation state is zero. W(CO)_6_ is a precursor molecule used in electron beam induced deposition (EBID) to produce well-defined tungsten-containing nanostructures [21–22]. Nanometre-thick films of surface-adsorbed W(CO)_6_ irradiated at 500 eV electron impact energy were analysed in situ by X-ray photoelectron spectroscopy (XPS), mass spectrometry and reflective absorption infrared spectroscopy (RAIRS) measurements [23]. These studies on electron-stimulated reactions of surface-adsorbed W(CO)_6_ molecules have shown contaminations by C and O due to incomplete ligand desorption yielding tungsten oxide and an enhanced degree of tungsten oxidation from the presence of co-adsorbed water. These contaminations are then incorporated into the carbonaceous matrix. Recently, we note ab initio molecular dynamics simulations of focused electron beam induced deposition (FEBID) precursor molecules adsorbed on fully and partially hydroxylated SiO_2_ surfaces [24]. Electron-induced reactions in FEBID processes are initiated by low-energy secondary electrons rather than the high-energy primary beam impinging on the surface where dissociative electron attachment (DEA) processes are relevant, although at those energies electron impact excitations yielding neutral dissociation are prevalent in detriment to DEA [25]. Gas-phase DEA studies in the electron energy range from 0 to 14 eV reported by Wnorowski et al. [26] revealed the strong dissociation character of W(CO)_6_, with no formation of bare W^−^ metal anions. Negative ion states of transition-metal hexacarbonyls have been obtained by electron transmission spectroscopy (ETS) with W(CO)_6_ attachment energies of 1.53, 2.46 and 4.26 eV [27]. We note electron impact ionisation studies on the appearance energies of bare tungsten hexacarbonyl [28], on the fragmentation pathways of W(CO)_6_ clusters [29] and on the complete ligand loss of weakly bound W(CO)_6_ dimer [30]. As far as neutral dissociation (ND) is concerned, Zlatar et al. [25] have reported on the relevance of electronically excited precursors yielding neutral fragmentation, although the products of ND processes appear to be more difficult to monitor than charged products in mass spectrometry. Therefore, detailed knowledge of the nature of the electronically excited states by experimental and theoretical methods are also demanded to assess the nature of the excited states from which estimates of ND cross sections can be obtained from electron energy loss spectroscopy. From the experimental point of view, such electron impact excitation spectra cannot be recorded with higher energy resolution than with optical spectra [31], making the latter an important tool to uncover features that may be not attained even in pseudo-optical conditions (high electron impact energy and low scattering angle) [32]. Another relevant aspect highlighted by Qi et al. [5] in the 30–160 nm wavelength region pertains to the similarity observed in the qualitative behaviour of neutral photodissociation and UV photoabsorption below the first ionisation energy, where CO ligand ejection occurs. Moreover, Venkataraman and co-workers [33] explored the photodissociation of W(CO)_6_ at 248 nm (ca. 5 eV) assigning the first CO elimination to a translational energy release process. As part of an ongoing effort to fully characterize the electron-induced fragmentation channels of W(CO)_6_, as needed for FEBID simulations, the purpose of the present work is to provide a high-resolution vacuum ultraviolet (VUV) absorption spectrum, representative of transition-metal carbonyl complexes for which unresolved spectral features remain to be solved, with a description as complete as possible of the electronic states.

In the next section we provide details on the experimental and theoretical methods used in this study for W(CO)_6_ followed by the results together with a discussion and comparison with previous data where possible. Finally, some concluding remarks that can be drawn from this study are given.

## Experimental and Theoretical Methods

### High-resolution vacuum ultraviolet photoabsorption

**Figure 1:**
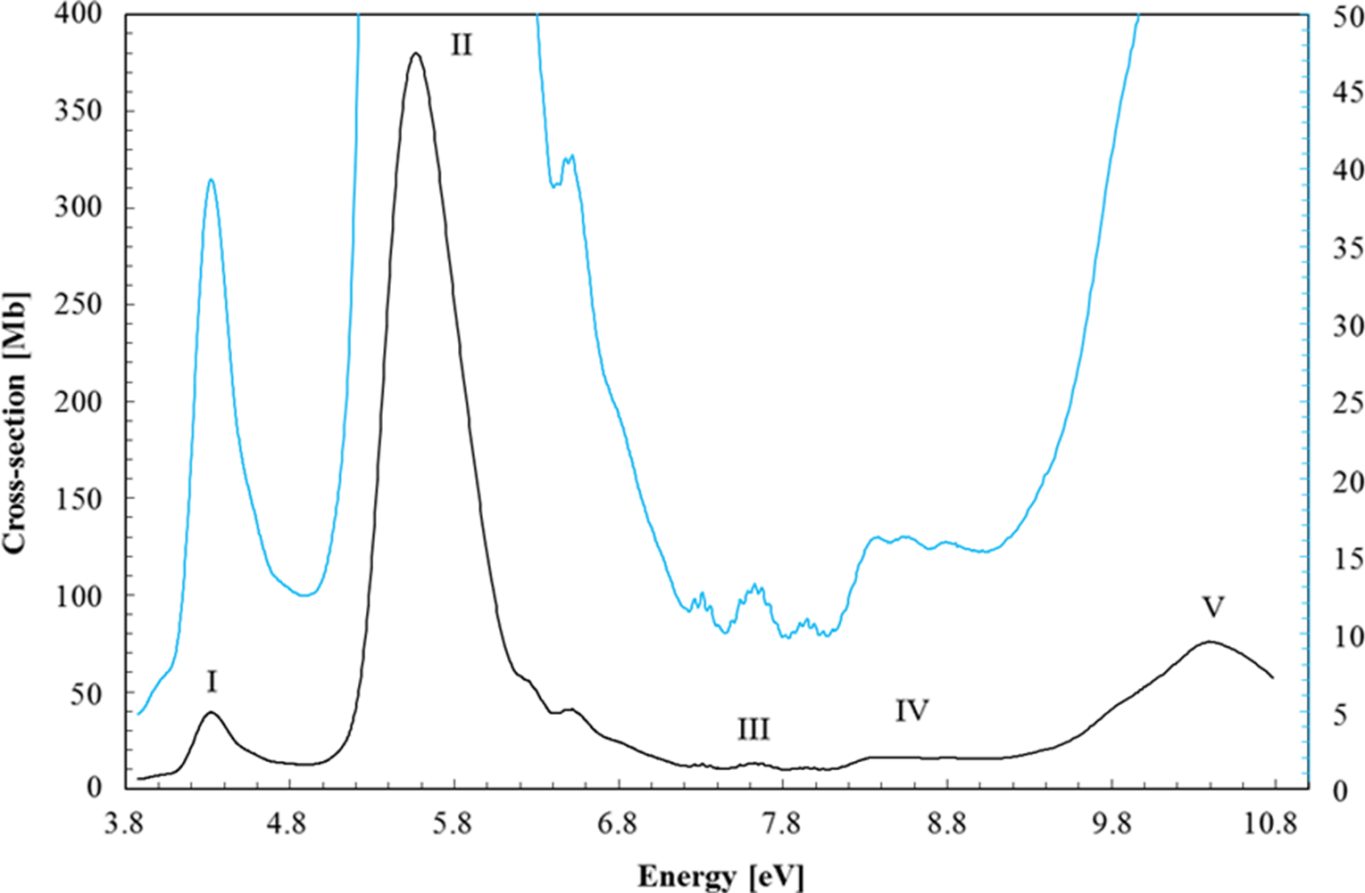
High-resolution VUV photoabsorption spectrum of W(CO)_6_ in the photon energy range of 3.9–10.8 eV. The blue curve has the (right) ordinate set to a maximum of 50 Mb to bring out the rich fine structure in the spectrum.

The high-resolution VUV photoabsorption studies of W(CO)_6_ in the photon energy range of 3.9–10.8 eV (Figure 1) were performed at the AU-UV beam line of the ASTRID2 synchrotron facility, Aarhus University, Denmark. The experimental setup has been described previously [31], with recent modifications reported in detail by Palmer et al. [34]. Briefly, an absorption gas cell is fitted with a heated 1 Torr full-scale Baratron capacitance manometer (Setra model 774) and a photo-multiplier tube (PMT) detector to measure the transmitted light intensity. In order to ensure that the data is free of any saturation effects, tungsten hexacarbonyl absorption cross sections were measured at an appropriate pressure with typical attenuations of less than 40%. The vacuum ultraviolet light exits the absorption cell through a MgF_2_ transmission window, which sets the lower limit of detectable light to 115 nm. A small gap between the PMT and the absorption cell is evacuated using a scroll pump to prevent any absorption of oxygen from the air for measurements below 200 nm (energies above 6.20 eV). For measurements above 220 nm, air is allowed into this gap to let oxygen absorb higher harmonics of light (at half the chosen wavelength) that may be passing through the cell.

Absolute photoabsorption cross sections (in units of megabarn, 1 Mb ≡ 10^−18^ cm^2^) are obtained using the Beer–Lambert attenuation law *I*_t_ = *I*_0_·exp(−*n*σ*x*), where *I*_t_ is the radiation intensity transmitted through the gas sample, *I*_0_ is the radiation intensity transmitted through the evacuated cell, *n* is the molecular number density of the sample gas, σ is the absolute photoabsorption cross section, and *x* is the absorption path length (here 15.5 cm). ASTRID2 is operated in a “top-up” mode, keeping the stored electron beam current (and thus the intensity for a given wavelength) quasi-constant, which is achieved by adding small amounts of current to the storage ring to make up for the constant beam decay. This procedure causes the beam current to vary by about 3% during a scan, with this effect being compensated for by a normalization of the data to an accurately determined beam current. The accuracy of the cross section is estimated to be better than ±5%. Only when the sample absorbs very weakly (*I*_0_ ≈ *I*_t_), does the error increase as a percentage of the measured cross section.

### Computational details

The structure of W(CO)_6_ has been fully optimized under the *O_h_* symmetry constraint at the density functional theory (DFT) level with B3LYP functional [35] with all electron and triple-ζ polarized basis sets in vacuum [36], leading to W–C and C–O bond lengths of 2.069 Å and 1.144 Å (1.141 Å from [15]), respectively. The metal bond length is slightly overestimated with respect to previous results yielding a W–C bond length between 2.047 and 2.063 Å [37]. However, the agreement is reasonable knowing that B3LYP overestimates metal–carbonyl bond lengths. The scalar relativistic effects have been included within the zero-order regular approximation (ZORA) [38]. The vertical spin-free absorption spectrum based on the 80 lowest excited states has been computed by means of time-dependent density functional theory (TDDFT) [39–40]. Spin–orbit coupling (SOC) effects have been applied as a perturbation to obtain the “spin–orbit” states. All calculations have been performed in vacuum with the ADF2013 code [41]. The computational protocol is based on our experience in the field of excited states of transition-metal complexes. In particular, the choice of B3LYP functional has been dictated by the good results obtained with 25% of XC in a number of transition-metal complexes characterized by a variety of excited states of different character. In contrast, CAM-B3LYP, which has been parametrized for small transition-metal compounds with different electronic properties more atomic-like, gives systematic overestimations of transition energies in the complexes [42–48].

### Tungsten hexacarbonyl sample

The sample used in the photoabsorption measurements was purchased from Sigma-Aldrich, with a stated purity of ≥99%. The sample was used as delivered.

## Results and Discussion

### Tungsten hexacarbonyl spectroscopy

**Figure 2:**
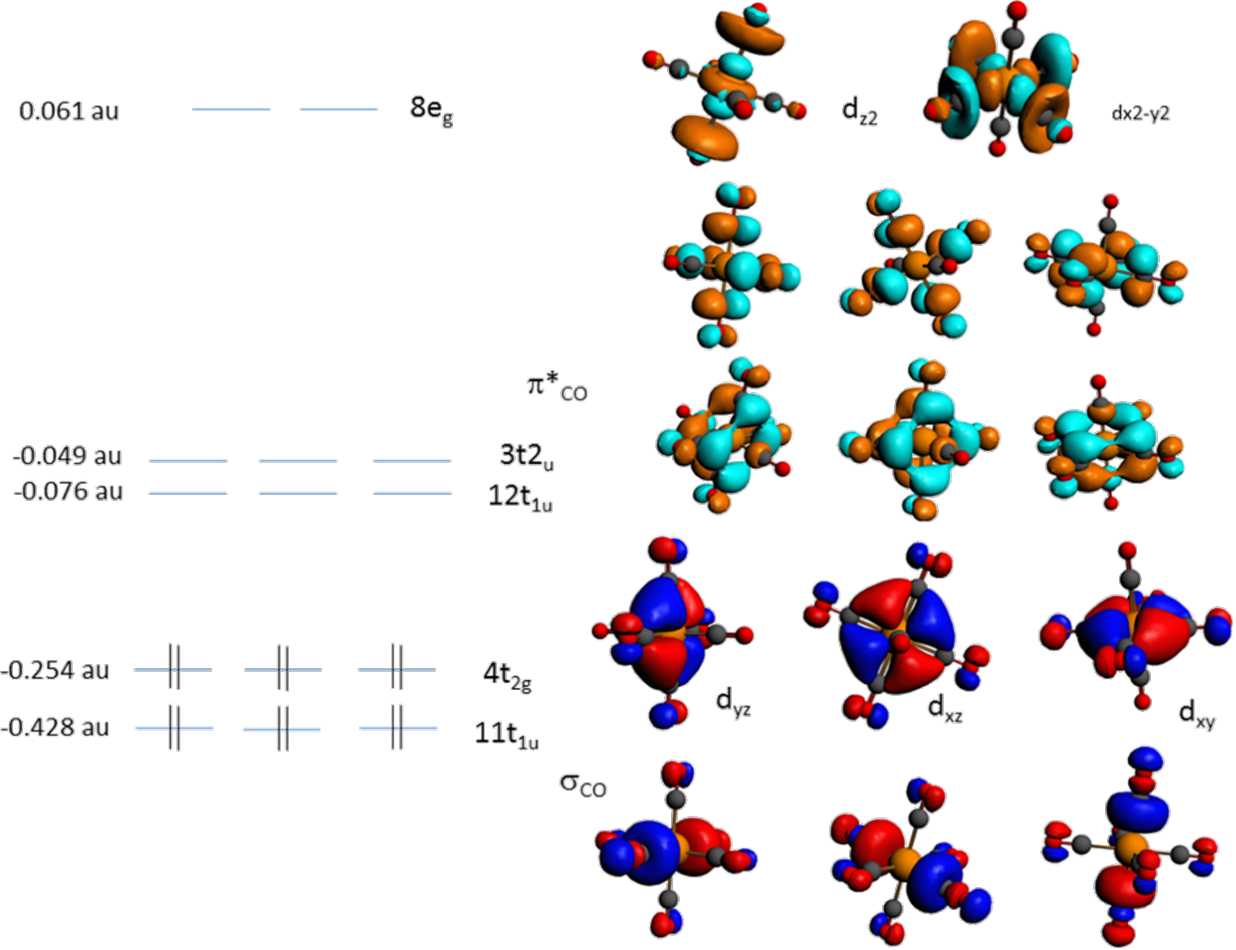
Valence Kohn–Sham orbitals of W(CO)_6_ in its electronic ground state.

W(CO)_6_ belongs to the *O_h_* point group with the calculated electronic configuration of the outermost valence orbitals for the ground state 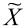 ^1^A_1g_ being (7e_g_)^4^ (3t_2g_)^6^ (1t_1g_)^6^ (11t_1u_)^6^ (4t_2g_)^6^, and only optically allowed ^1^A_1g_→^1^T_1u_ transitions. The analysis of the ground-state Kohn–Sham (KS) orbitals (Figure 2 and Figure S1 in Supporting Information File 1) shows that the highest occupied molecular orbital (HOMO), 4t_2g_, has metal *d*_π_ character, the second-highest molecular orbital (HOMO−1), 11t_1u_, has σ_CO_ character, the third-highest (HOMO−2), 1t_1g_, and the fourth-highest occupied molecular orbitals (HOMO−3), 3t_2g_, have CO character. The lowest unoccupied molecular orbital (LUMO), 12t_1u_, and (LUMO+1), 3t_2u_, have mainly π*_CO_ antibonding character, while (LUMO+2), 13t_1u_, and (LUMO+3), 2t_1g_, have π*_CO_ and σ*_CO_ character, respectively.

Interpretation of the experimental data and results from quantum chemical calculations of the lowest electronic states with spin–orbit coupling (SOC) are summarised in Table 1. Singlet and triplet excited state energies without SOC are reported in Table S1 and Table S2 of Supporting Information File 1. The calculated transition energies reported in Table 1 are generally within 0.1–0.2 eV when compared with the experiment, with the exception of the strongest absorption band where the difference amounts to an overestimation of 0.5 eV. Nonetheless, this level of accuracy is reasonable for describing the VUV photoabsorption features. The TDDFT absorption spectrum of W(CO)_6_ without SOC is depicted in Figure S2 of Supporting Information File 1.

**Table 1:** TDDFT vertical transition energies and associated oscillator strengths (*f*_0_) of the low-lying “spin–orbit” excited states of W(CO)_6_ and assignment of the experimental spectrum.

energy^a^ (eV)	cross section (Mb)	band	state	composition in “spin-free” states^b^	calculated energy (eV)	*f*_0_	character

<4.0			1T_1u_	72% 1^3^T_1u_ 19% 1^3^E_u_	3.458	3.7·10^−4^	MLCT
			2T_1u_	40% 1^3^E_u_ 33% 1^3^T_2u_ 26% 1^3^T_1u_	3.596	1.2·10^−4^	MLCT
			3T_1u_	59% 1^3^T_2u_ 40% 1^3^Eu	3.656	4.1·10^−5^	MLCT
4.317	39.4	I	4T_1u_	91% 1^1^T_1u_	4.11	0.02	MLCT
			6T_1u_	40% 2^3^T_1u_ 38% 1^3^A_1u_ 15% 2^3^E_u_	4.309	0.002	MLCT/IL
5.590	377.7	II	9T_1u_	99% 2^1^T_1u_	6.106	0.93	MLCT
7.630	13.2	III	10T_1u_	99% 3^3^T_1u_	7.965	3.2·10^−5^	IL
8.35(5)	16.2	IV	11T_1u_	27% 3^3^E_u_ 25% 3^1^T_1u_ 24% 4^3^T_1u_ 23% 3^3^T_2u_	8.146	0.009	MLCT
			12T_1u_	38% 3^3^T_2u_ 37% 4^3^T_1u_ 13% 3^1^T_1u_ 12% 3^3^E_u_	8.264	0.005	MLCT
			13T_1u_	36% 3^3^E_u_ 32% 4^3^T_1u_ 19% 3^3^T_2u_ 13% 3^1^T_1u_	8.329	0.004	MLCT
			14T_1u_	49% 3^1^T_1u_ 25% 3^3^E_u_ 20% 3^3^T_2u_	8.340	0.017	MLCT
10.375	75.5	V	21T_1u_	85% 4^1^T_1u_ 14% 6^3^T_1u_	10.011	0.057	IL
			22T_1u_	79% 6^3^T_1u_ 14% 4^1^T_1u_	10.036	0.009	IL
			24T_1u_	64% 7^3^T_1u_ 31% 5^1^T_1u_	10.211	0.012	IL
			25T_1u_	59% 5^1^T_1u_ 31% 7^3^T_1u_	10.242	0.021	IL
			27T_1u_	37% 3^3^A_1u_ 30% 7^1^T_1u_ 17% 6^1^T_1u_	10.456	0.027	IL
			30T_1u_	60% 6^1^T_1u_ 20% 7^1^T_1u_	10.524	0.017	IL
			32T_1u_	32% 7^1^T_1u_ 27% 7^3^E_u_ 20% 3^3^A_1u_	10.557	0.027	IL
			33T_1u_	43% 7^3^E_u_ 39% 8^3^E_u_	10.632	0.004	IL
			34T_1u_	52% 8^3^E_u_	10.701	0.008	IL
			36T_1u_	57% 8^1^T_1u_ 22% 9^3^T_1u_	10.867	0.026	SBLCT
			37T_1u_	38% 8^1^T_1u_ 33% 9^3^T_1u_	10.889	0.021	SBLCT

^a^The last decimal of the energy value is given in parenthesis for these less-resolved features. ^b^According to the labels of the states reported in Table S1 and Table S2 of Supporting Information File 1.

### Valence transitions

The measured high-resolution VUV photoabsorption spectrum is presented in Figure 1, in the photon energy range from 3.9 to 10.8 eV, and the proposed assignments are summarised in Table 1 based on the vibrational spectra of Amster et al. [18], Broquier et al. [19] and the infrared data of Jones [20]. The main features have been identified as bands I to V and their main characteristics are discussed below, with a complete overview and assignment of the electronic structure. The TDDFT calculations with SOC predict an important contribution of the triplet states for bands III and IV as discussed further in the next sections.

#### A. Low-lying electronic states (below 4.0 eV)

The temperature-dependent absorption studies of Beach and Gray [2] of W(CO)_6_ in a solvent report a weak band at 3.794 eV, which was assigned to a spin-forbidden d→d transition. In contrast, our calculations do not provide evidence for the presence of low-lying metal-centred (MC) states in this period 6 transition-metal carbonyl complex, as it is the case in the period 4 analogue Cr(CO)_6_ [49]. The electron energy loss spectrum under non-dipolar conditions of Koerting and co-workers [6] reports a feature at 3.75 eV, which was assigned to a symmetry-forbidden feature. Rosa and co-workers [15] have proposed that in the region of 3.5–4.0 eV spin-allowed but symmetry-forbidden charge-transfer transitions as well as spin-forbidden but orbital-allowed ^1^A_1g_→a,b^3^T_1u_ transitions occur. The present calculations including SOC effects show that three “spin–orbit” states, namely 1T_1u_, 2T_1u_ and 3T_1u_ of metal-to-ligand charge transfer (MLCT) character, which are composed essentially of 1^3^T_1u_, 1^3^E_u_ and 1^3^T_2u_ states, contribute to this region (Table 1 and Table S1 in Supporting Information File 1).

#### B. Band I (3.9–4.8 eV/330–260 nm)

**Figure 3:**
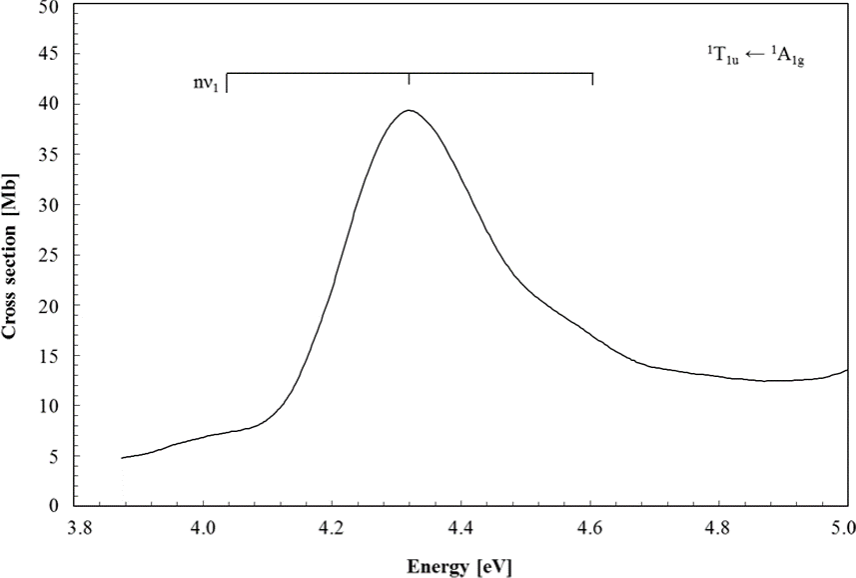
High-resolution VUV photoabsorption spectrum of W(CO)_6_ in the photon energy range of 3.9–5.0 eV. See text for details on the assignments.

The photoabsorption spectrum in the energy region of 3.9–4.8 eV is shown in Figure 3 and the proposed assignments are summarised in Table 2.

The main absorption feature peaking at 4.317 eV with a local cross section of 39.4 Mb is assigned to the “spin–orbit” 4T_1u_ state composed mainly of the 1^1^T_1u_←1^1^A_1g_ transition involving the contribution from 12t_1u_←4t_2g_ (0.66) and 3t_2u_←4t_2g_ (0.33) of MLCT character and calculated to be at 4.119 eV (Table 1) in agreement with previous assignments. Indeed, this band has been identified by Gray and Beach [1] to MLCT at 4.328 eV, 4.32 eV by Koerting et al. [6], 4.36 eV by Cooper et al. [7], 4.30 eV by Pradeep [8] and 4.336 eV by Trogler and co-workers [4]. Rosa et al. [15] employing a combined TD-DFT/ZORA method, but without SOC effects, obtained a transition energy at 3.80 eV. Another interesting aspect is that ETS studies have reported a negative ion state at 4.26 eV [27], which can certainly be related to the main feature in the energy region of 3.9–4.8 eV.

The (0–0) origin band is not pronounced in the spectrum, although tentatively estimated to be at 4.04(6) eV (Table 2), according to the assignment on the vibrational structure reported by Trogler and co-workers [4]. The assignments of Gray and Beach [1–2] were based on electronic transitions at 4.039 and 4.609 eV assigned to ^1^T_1g_←^1^A_1g_ and ^1^T_2g_←^1^A_1g_ and expected to be weak because of a dipole-forbidden nature. Note that the electron energy loss spectroscopy data only reports the ^1^T_2g_←^1^A_1g_ transition at 4.54 eV.

**Table 2:** Proposed vibrational assignments in the 4.0−9.0 eV absorption bands of W(CO)_6_. (b) broad structure; (s) shoulder structure; (w) weak feature. The last decimal of the energy value is given in brackets for these less-resolved features).

energy (eV)	assignment	Δ(ν′_2_) (eV)	Δ(ν′_1_) (eV)

**band I**			

4.04(6) (b,s)	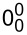	—	—
4.317	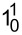	—	0.271
4.60(2) (s)	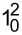	—	0.285

**band II**			

6.15(6) (s)	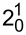	—	—
6.19(6) (s)	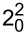	0.040	—
6.24(3) (s)	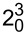	0.047	—
6.29(7) (s)	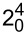	0.054	—
6.42(4) (w)	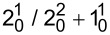	—	0.228
6.471	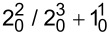	0.047	0.228
6.515	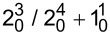	0.044	0.218
6.567	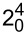	0.052	—
6.79(4) (b)	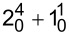	—	0.227

**band III**			

7.16(7) (w,b)	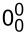	—	—
7.208	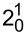	0.041	—
7.259	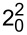	0.051	—
7.315	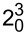	0.056	—
7.358	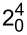	0.043	—
7.41(1) (b)	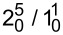	0.053	0.244
7.496	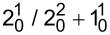	—	0.237
7.542	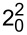	0.046	—
7.583	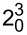	0.041	—

**band III**			

7.630	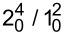	0.047	0.219
7.672	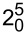	0.042	—
7.720	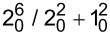	0.048	0.224
7.76(4) (s)	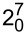	0.044	—
7.80(8) (b)	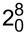	0.044	—
7.813	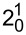	—	—
7.862	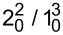	0.049	0.232
7.907	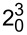	0.045	—
7.948	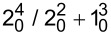	0.041	0.228
7.994	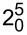	0.046	—
8.03(5) (b)	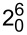	0.041	—

**Band IV**			

8.098	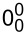	—	—
8.16(0) (s)	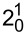	0.062	—
8.21(1) (s)	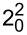	0.051	—
8.26(0) (s)	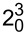	0.049	—
8.30(4) (s)	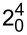	0.044	—
8.35(5) (b)	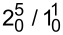	0.051	0.257
8.38(9) (b)	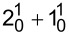	—	0.178
8.574	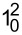	—	0.219
8.60(4) (s)	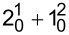	—	0.215
8.79(9) (b)	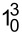	—	0.225
8.85(6) (w)	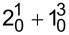	—	0.252

The calculations of Rosa et al. [15] assign this feature to the close-lying ^1^A_1g_→a^1^T_1g_ and ^1^A_1g_→a^1^T_2g_ symmetry-forbidden charge-transfer transitions. However, on the basis of the present calculations it seems that these symmetry-forbidden transitions do not participate in this band. In contrast, the mixed MLCT/intra-ligand (IL) 6T_1u_ “spin–orbit” state calculated at 4.309 eV, with a modest oscillator strength (0.002) and composed essentially of 2^3^T_1u_, 1^3^A_1u_ and 2^3^E_u_ (Table 1), should contribute significantly. The present assignments are therefore based on a weak vibrational progression of the CO stretching mode ν_1_, with 0.263 eV (2124 cm^−1^) in the ground state [20], although an average of 0.278 eV is observed in Table 2 and is certainly due to the broad and shoulder nature of the experimental features.

#### C. Band II (5.0–7.0 eV/248–177 nm)

**Figure 4:**
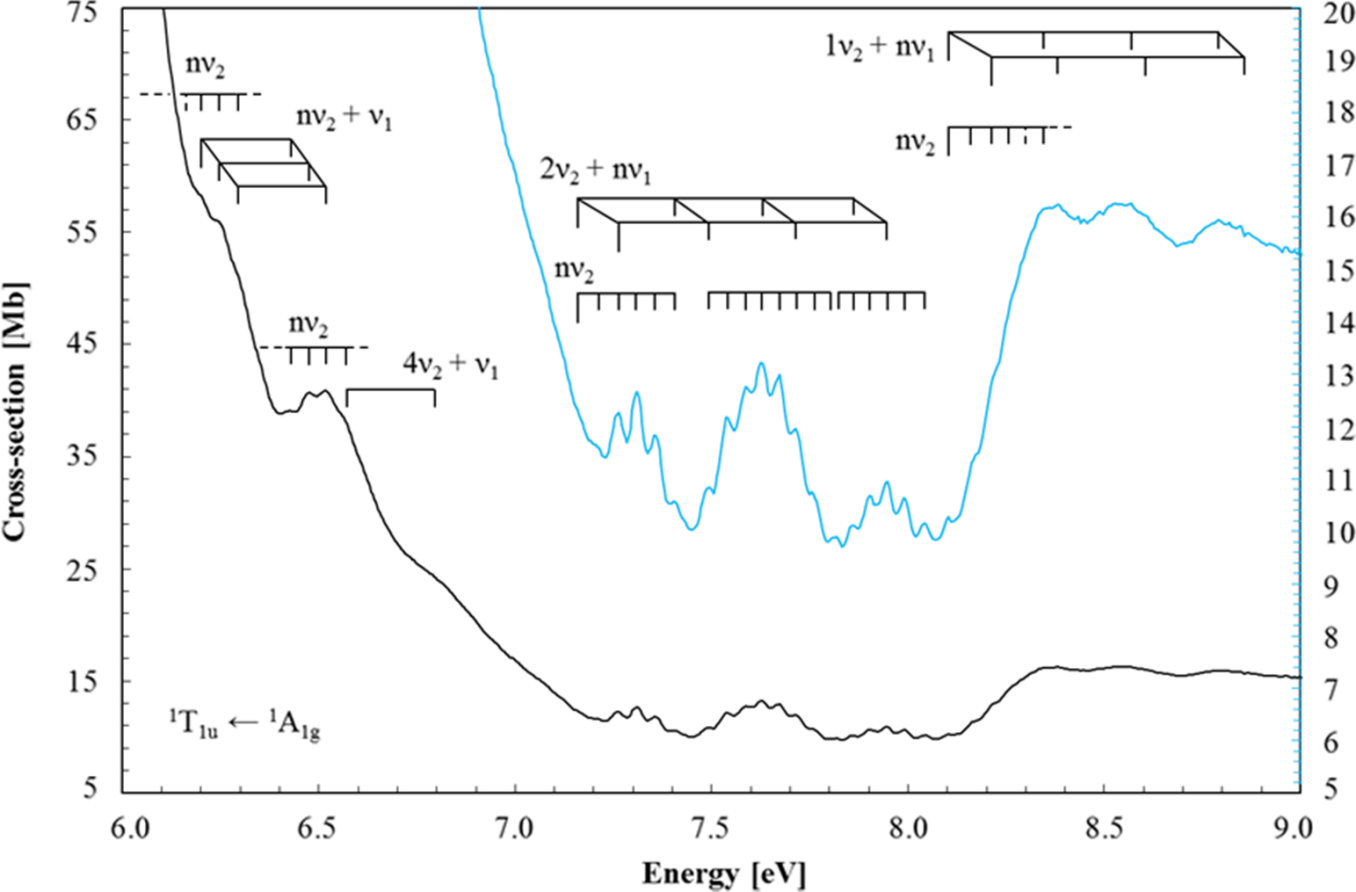
High-resolution VUV photoabsorption spectrum of W(CO)_6_ in the photon energy range of 6.0–9.0 eV. See text for details on the assignments.

The photoabsorption spectrum of band II is shown in Figure 1 and our spectral assignments are shown in Table 2 and Figure 4. This is the most intense band within the photon energy range studied and corresponds mostly to the 2^1^T_1u_←1^1^A_1g_ MLCT transition involving the contribution from (3t_2u_←4t_2g_) (0.64) and (12t_1u_←4t_2g_) (0.31) (Table 1) calculated at 6.106 eV with a large oscillator strength (0.93), and peaking in the experimental spectrum at 5.590 eV. This electronic excitation value is consistent with Gray and Beach [1–2] at 5.535 eV, Iverson and Russell [3] at 5.56 eV, Trogler et al. [4] at 5.391 eV, Koerting et al. [6] and Cooper et al. [7] at 5.5 eV, Pradeep [8] at 5.4 eV and Rosa et al. [15] at 5.84 eV. The main absorption feature has a local cross section of 377.7 Mb compared to 35.3 Mb from ref. [3] and on the high-energy side shows fine structure reminiscent of vibrational progressions involving the predominantly CO stretching mode *ν*_1_, and WC stretching mode *ν*_2_ (Table 2). The *ν*_1_-mode excited state frequency appears to drop by about 53 meV (to 0.225 eV) from Band I and the *ν*_2_-mode has an average excitation energy of 0.047 eV, which may be compared to 0.052 eV (392 cm^-1^) in the ground state [4,20]. Gray and Beach [1] assigned features at 6.263 and 6.526 eV to t_2g_(π)→t_2g_(π*) transitions, whereas Koerting et al. [6] at 6.24 and 6.54 eV report them as ligand-field (LF) ^1^A_1g_→^1^A_1g_ symmetry forbidden transitions.

It is interesting to note that in [9] an identical assignment was proposed for the feature at 6.51 eV while the calculation of Rosa et al. [15] only predicts LF transitions at higher energies, i.e., 7.33 and 7.44 eV but of T_1g_ and T_2g_ character. Our calculations do not support these assignments but rather singlet-to-triplet excitations of Rosa’s character (T_1g_ and T_2g_) but at lower energies (see Table S2, Supporting Information File 1).

The photodissociation mechanisms of W(CO)_6_ have been investigated in a crossed laser–molecular beam study at 248 nm (ca. 5 eV), exploring the velocity distributions of the nascent photofragment yields [33]. A detailed analysis of the TOF photofragment distributions has provided a reliable description of the photodissociation mechanism for all six CO elimination steps required to produce the bare metal atom [33]. The single-photon and multiphoton processes yield sequential metal-to-ligand bond breaking with CO translational energy distributions assigned to direct and statistical dissociation, where the repulsive energy release in the first CO elimination step is expected to prevail in the condensed phase.

#### D. Band III (7.0–8.0 eV/177–155 nm)

The photoabsorption spectrum of band III can be seen in Figure 1 and Figure 4. Within this region we find the richest fine structure. The electronic absorption band that is the weakest over the whole energy range peaks at 7.630 eV with a local maximum cross section of 13.2 Mb in contrast to 7.65 eV and 1.56 Mb from Iverson and Russell [3]. It corresponds to a 3^3^T_1u_←1^1^A_1g_ transition involving the contribution from 12t_1u_←1t_1g_ (0.27), 3t_2u_←1t_1g_ (0.16), (12t_1u_←3t_2g_ (0.16) and 3t_2u_←3t_2g_ (0.11) calculated at 7.965 eV (Table 1). The calculation suggests that the transition is due to IL electronic excitation. Koerting et al. [6] have assigned the feature at 7.66 eV to the ^1^A_1g_→^1^A_1g_ symmetry-forbidden transition, where Cooper et al. [7] reported this at 7.48 eV. Transitions to singlet states from the ground state are symmetry-forbidden, but can gain some intensity through vibronic coupling [50–51]. Although our calculations do not predict an excitation to a singlet state for band III, the considerable rich fine structure may be responsible for making this absorption band, although weak, noticeable. Here, we report for the first time a comprehensive assignment of the features that involve excitation of the CO stretching mode ν_1_, and WC stretching mode ν_2_ (see Figure 4 and Table 2). The (0–0) origin band is tentatively estimated to be at 7.16(7) eV (Table 2) and the average energies of modes ν_1_ and ν_2_ are 0.231 and 0.046 eV, respectively. Rosa et al. [15] calculated the singlet LF states c^1^T_1g_ and d^1^T_2g_ at 7.33 and 7.44 eV, respectively, and the corresponding triplet states c^3^T_1g_ and d^3^T_2g_ at 7.09 and 7.19 eV, respectively. Our calculations predict two ^3^MC states, namely ^3^T_2g_ and ^3^T_1g_ calculated at 7.08 and 6.96 eV, respectively (Table S2 of Supporting Information File 1).

#### E. Band IV (8.0–9.0 eV/155–138 nm)

The photoabsorption spectrum of band IV is presented in Figure 4, with the spectral assignments being contained in Table 2. It has been assigned to a transition involving the contribution of (0.49) 3^1^T_1u_/(0.25) 3^3^E/(0.20) 3^3^T_2u_ from the electronic ground state (13t_1u_←4t_2g_) (0.92) (Table 1) calculated at 8.340 eV, peaking at 8.35(5) eV with a maximum cross section of 16.2 Mb. Three other transitions calculated at 8.146, 8.264 and 8.329 eV composed essentially of 3^3^E_u_, 3^3^T_2u_ and 3^3^T_1u_ (Table 1) may contribute to this band. The vacuum ultraviolet spectrum of Iverson and Russell report a value at 8.43 eV and a cross section value of 1.7 Mb [3]. The pseudo-photon measurements of Koerting et al. [6] and Cooper et al. [7] report values at 8.38 and 8.25 eV. The electron energy loss measurements assigned this transition to a symmetry-forbidden ^1^A_1g_→^1^A_1g_ transition [6–7], in contrast to our assignment. The main absorption features within this band have been identified as having MLCT character. The origin of the band is placed at 8.098 eV (Table 2) and the fine structure has been assigned as combinations of the CO stretching mode ν_1_ (average value of 0.224 eV), and WC stretching mode ν_2_ (average value of 0.051 eV). The lowest-lying vertical ionisation energy of tungsten hexacarbonyl has been reported by Lloyd and Schlag [11] at 8.38 eV and the significant rise in the absorption spectrum signal above this energy may accommodate contributions from the ionic state.

#### F. Band V (above 9 eV)

Tungsten hexacarbonyl displays a broad and structureless feature in this energy region (Figure 1). The main feature is attributed to an IL transition, peaking at 10.375 eV and with a local maximum cross section value of 75.5 Mb. Electron energy loss spectroscopy data reported values at 9.75 eV [6] and 10.3 eV [7], whereas the photoabsorption data of Qi et al. [5] reveals a feature at 10.06 eV followed by a CO vibrational structure with several quanta being excited. Koerting et al. [6] reported that features at 9.8 and 10.4 eV may be due to Rydberg transitions or IL transitions to super-excited states. Several transitions calculated between 10.011 and 10.889 eV with reasonable oscillator strengths may contribute to band V (Table 1). The most intense ones involve the upper ^1^T_1u_ states of IL or sigma-bond-to-ligand charge transfer (SBLCT) character, whereas ^3^T_1u_ and ^3^E_u_ states may also contribute significantly. In some states the singlet/triplet mixing may be rather important such as in 27T_1u_ and 32T_1u_ for instance.

## Conclusion

We have presented a comprehensive investigation of the experimental and theoretical electronic transitions in tungsten hexacarbonyl, W(CO)_6_. Our high-resolution synchrotron photoabsorption measurements allowed for the identification of previously unresolved experimental features for the first time. The level of accuracy of our relativistic calculations has allowed the reassessment and reassignment of some states, particularly those previously explored below 8 eV. However, despite a reasonable agreement between the TDDFT and VUV absorption spectra we should be aware that the description of double excited states or diffuse Rydberg states is not accessible at the present level of calculation due to the limitation of TDDFT to single excitations. Unfortunately, the use of large diffuse basis sets necessary to correctly describe Rydberg states is beyond the current computational capabilities because of numerical problems due to near-linear dependencies in the basis sets. The combined experimental/theoretical investigation appears relevant to assess the role of W(CO)_6_ low-lying electronic states that can lead to dissociation. Thus, a reasonable assumption is given that the electronic excitation of tungsten hexacarbonyl leads to direct and statistical bond breaking, the former reminiscent of a repulsive dissociation character by the translational energy distribution of the first CO ligand, the latter correctly modelled by statistical product energy distributions [33].

## Supporting Information

File 1 Additional computational data.
