# Supplementary material for: Comprehensive investigation of the electronic excitation of W(CO)6 by photoabsorption and theoretical analysis in the energy region from 3.9 to 10.8 eV
Source: Beilstein J Nanotechnol. 2017 Oct 23;8:2208–18. doi: 10.3762/bjnano.8.220 (PMC5669226; doi:10.3762/bjnano.8.220)
Supplement: File 1 — Additional computational data. [file Beilstein_J_Nanotechnol-08-2208-s001.pdf]

Supporting Information

for

Comprehensive investigation of the electronic excitation of $W(CO)_6$ by photoabsorption and theoretical analysis in the energy region from 3.9 to 10.8 eV

Mónica Mendes[1], Khrystyna Regeta[1], Filipe Ferreira da Silva[1], Nykola C. Jones[2], Søren Vrønning Hoffmann[2], Gustavo García[3], Chantal Daniel*[,4] and Paulo Limão-Vieira*[,1]

Address: [1]Laboratório de Colisões Atómicas e Moleculares, CEFITEC, Departamento de Física, Universidade NOVA de Lisboa, 2829-516, Caparica, Portugal; [2]ISA, Department of Physics and Astronomy, Aarhus University, Ny Munkegade 120, DK-8000, Aarhus C, Denmark [3]Instituto de Física Fundamental, Consejo Superior de Investigaciones Científicas (CSIC), Serrano 113-bis, 28006 Madrid, Spain and [4]Laboratoire de Chimie Quantique, Institut de Chimie Strasbourg, UMR7177 CNRS/Université de Strasbourg 1 Rue Blaise Pascal BP296/R8, F-67008 Strasbourg, France

Email: Paulo Limão-Vieira* - plimaovieira@fct.unl.pt; Chantal Daniel* - c.daniel@unistra.fr;

* Corresponding author

Additional computational data

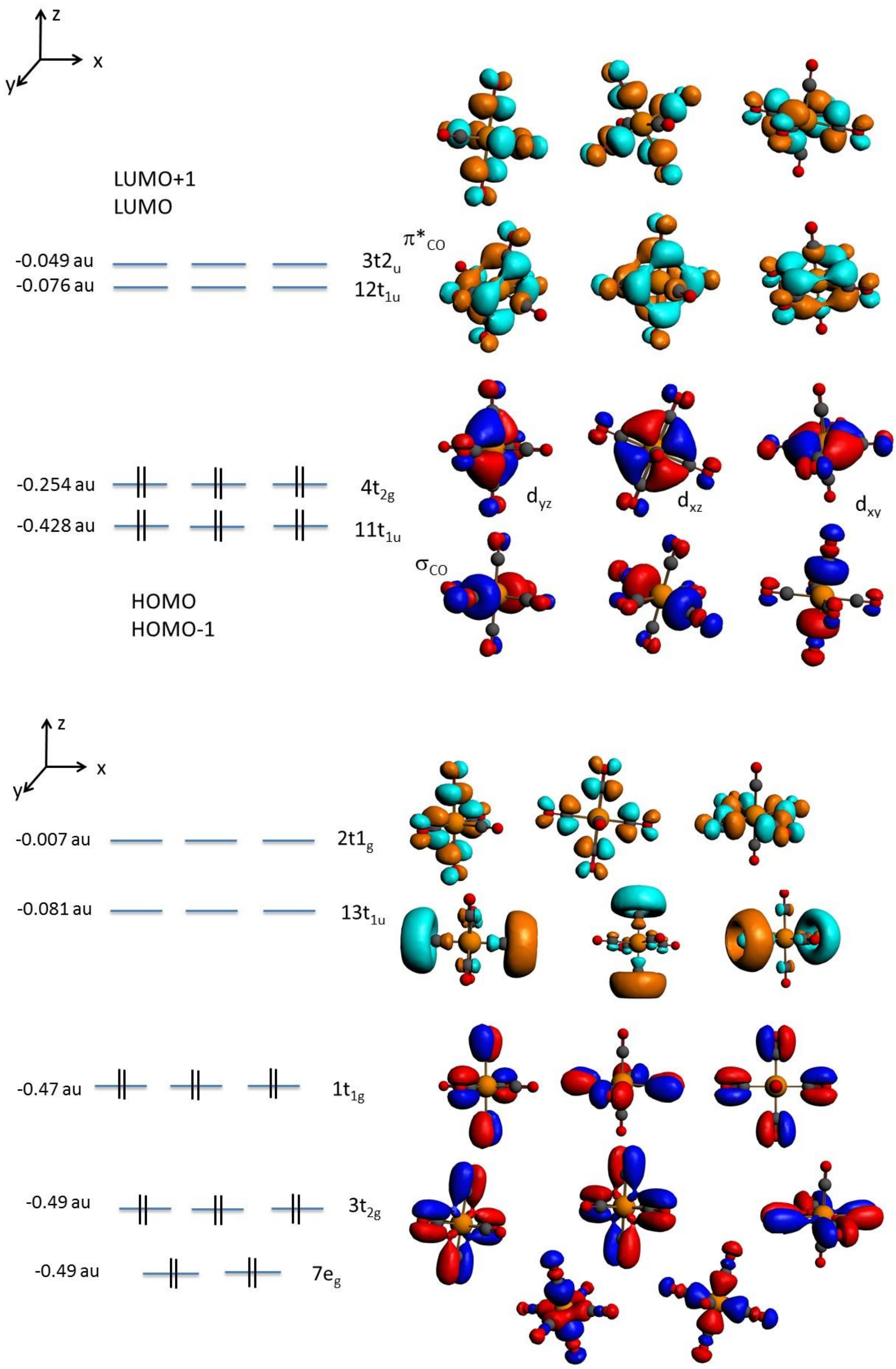

Figure S1: Kohn–Sham orbitals of $W(CO)_6$ (O_h symmetry) involved in major electronic transitions.

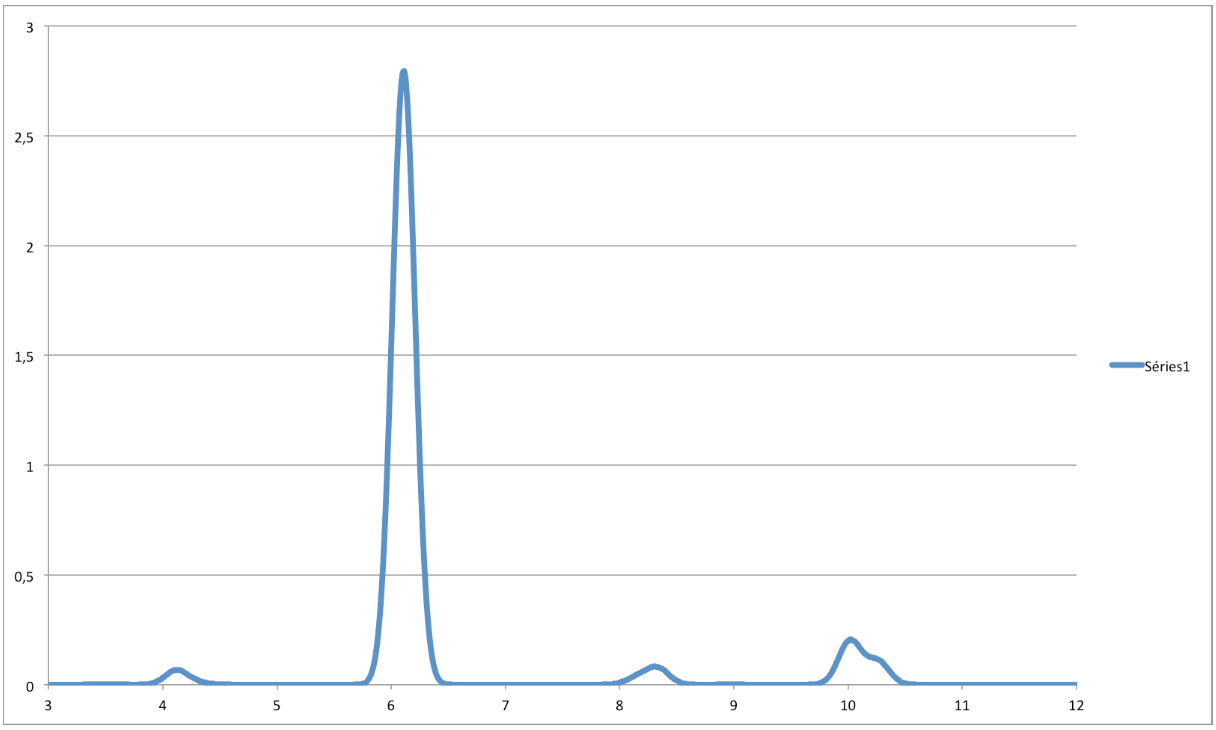

Figure S2: TDDFT spectrum of $W(CO)_6$ without spin–orbit coupling.

Table S1: Transition energies (in eV), oscillator strengths (>10^{-3}) and character of the lowest singlet states without spin–orbit coupling.

state	transition energy (eV)	oscillator strength	character
1^1T_{1u}	4.12	0.024	$^1MLCT_{CO}$
2^1T_{1u}	6.10	0.93	$^1MLCT_{CO}$
3^1T_{1u}	8.28	0.035	$^1MLCT_{CO}$
1^1T_{1g}	7.21	0.0	^1MC
1^1T_{2g}	7.43	0.0	^1MC
4^1T_{1u}	10.02	0.067	^1IL
5^1T_{1u}	10.26	0.035	^1IL
7^1T_{1u}	10.52	0.083	^1IL
8^1T_{1u}	10.88	0.042	^1IL
9^1T_{1u}	10.97	0.042	^1IL
10^1T_{1u}	11.12	0.011	^1IL
11^1T_{1u}	11.21	0.017	^1IL
12^1T_{1u}	11.52	0.001	^1IL

Table S2: Transition energies (in eV) and character of the lowest triplet states without spin-orbit coupling.

state	transition energy (eV)	character
1^3A_{2u}	3.46	$^3MLCT_{CO}$
1^3T_{1u}	3.46	$^3MLCT_{CO}$
1^3T_{2u}	3.59	$^3MLCT_{CO}$
1^3E_u	3.60	$^3MLCT_{CO}$
2^3E_u	4.27	$^3MLCT_{CO}$
1^3A_{1u}	4.37	^3IL
2^3T_{1u}	4.38	$^3MLCT_{CO}$
2^3T_{2u}	4.42	$^3MLCT_{CO}$
1^3E_g	5.28	$^3MLCT_{CO}$
1^3A_{2g}	5.39	$^3MLCT_{CO}$
1^3T_{2g}	5.54	$^3MLCT_{CO}$
1^3T_{1g}	5.62	$^3MLCT_{CO}$
1^3A_{1g}	5.74	$^3MLCT_{CO}$
2^3E_g	6.17	$^3MLCT_{CO}$
2^3T_{2g}	6.26	$^3MLCT_{CO}$
3^3T_{2g}	6.34	$^3MLCT_{CO}$
2^3T_{1g}	6.53	$^3MLCT_{CO}$
3^3T_{1g}	6.96	^3MC
4^3T_{2g}	7.08	^3MC
2^3A_{1g}	7.69	^3IL
5^3T_{2g}	7.72	^3IL
4^3T_{1g}	7.75	^3IL
3^3E_g	7.94	^3IL
3^3T_{1u}	7.97	^3IL
4^3T_{1u}	8.22	$^3MLCT_{CO}$
3^3T_{2u}	8.22	$^3MLCT_{CO}$
2^3A_{2u}	8.26	$^3MLCT_{CO}$
3^3E_u	8.27	$^3MLCT_{CO}$
4^3E_g	8.31	^3IL
6^3T_{2g}	8.60	^3IL
2^3A_{2g}	8.66	^3IL
4^3E_u	8.72	^3IL
4^3T_{2u}	8.74	$^3MLCT_{CO}$
3^3A_{1g}	8.75	^3IL
3^3A_{2u}	8.84	^3IL
5^3E_g	8.93	^3IL
5^3T_{2u}	8.94	^3IL
5^3T_{1g}	8.95	^3IL
5^3T_{1u}	9.00	^3IL
7^3T_{2g}	9.25	^3IL
2^3A_{1u}	9.26	^3IL
6^3E_g	9.41	^3IL
5^3E_u	9.45	^3IL

3^3A_{2g}	9.46	^{3}IL
6^3T_{1g}	9.51	^{3}IL
6^3T_{1u}	10.04	^{3}IL
6^3T_{2u}	10.06	^{3}IL
8^3T_{2g}	10.06	^{3}IL
7^3T_{1g}	10.07	^{3}IL
8^3T_{1g}	10.14	^{3}MC
9^3T_{2g}	10.20	^{3}MC
7^3T_{1u}	10.22	^{3}IL
7^3T_{2u}	10.43	^{3}IL
4^3A_{1g}	10.45	$^3MLCT_{CO}$
6^3E_u	10.48	^{3}IL
10^3T_{2g}	10.49	^{3}IL
7^3E_g	10.51	^{3}IL
8^3T_{1u}	10.51	^{3}IL
